# Assessment of The Diagnostic Capability of Serum Creatine Phosphokinase and Its Isoenzyme in Ectopic Pregnancy

**Published:** 2012-12-17

**Authors:** Attaollah Ghahiri, Aidin Moshreffar, Aida Najafian, Mojdeh Ghasemi, Hatav Ghasemi Tehrani

**Affiliations:** 1 Department of Obstetrics and Gynecology, Al Zahra Hospital, Isfahan University of Medical Sciences, Isfahan, Iran; 2 Department of Obstetrics and Gynecology, Hormozgan University of Medical Sciences, Bandar Abbas, Iran; 3 Research Office, Shahid Beheshti Hospital, Isfahan University of Medical Sciences, Isfahan, Iran

**Keywords:** Tubal Pregnancy, CPK, CPK-MB

## Abstract

**Background:**

Our goal was to assess the diagnostic value of creatine phosphokinase (CPK) and its isoenzyme CPK- muscle brain (MB) in ectopic pregnancy (EP) in order to locate a simpler diagnostic approach for EP.

**Materials and Methods:**

This was a prospective study that performed consecutive sampling for 20 months in two major hospitals in Isfahan, Iran. All pregnant patients in their first trimester of gestation that presented with complaints of vaginal bleeding, abdominal pain, or both enrolled in this study. Blood sampling was performed for laboratory analyses (CPK, CPK-MB). After their diagnosis was established, patients were put in either the EP or non-EP group. We used SPSS software version 10 for data analysis, diagnostic parameters were determined, and a relative operating characteristic (ROC) curve was plotted for each biochemical marker.

**Results:**

A total of 106 patients, 53 in the EP group and 53 in the non-EP group enrolled in this study. The results for CPK were as follows: sensitivity (69.81%), specificity (64.15%), positive predictive value (PPV; 66.07%), negative predictive value (NPV; 68%), positive likelihood ratio (PLR) (1.95), and negative likelihood ratio (NLR) (0.49). The results for CPK-MB were: sensitivity (71.7%), specificity (56.6%), PPV (62.29%), NPV (66.7%), PLR (1.65), and NLR (0.5). The area under the ROC curve for CPK was 0.692 and for CPK-MB it was 0.647.

**Conclusion:**

Although we have observed a significant elevation in CPK and CPK-MB serum levels in EP, transvaginal ultrasound (TVS) is still the better diagnostic tool for EP.

## Introduction

Ectopic pregnancy (EP) occurs when the developing blastocyst becomes implanted at a site other than the endometrium of the uterine cavity. The most common extra-uterine location is the fallopian tube, which accounts for 98% of all ectopic gestations. It is important to remember that hemorrhage from EP is still the leading cause of pregnancy-related maternal death in the first trimester and accounts for 4-10% of all pregnancy-related deaths, despite improved diagnostic methods that lead to earlier detection and treatment. The development of a sensitive, specific radioimmunoassay for human chorionic gonadotropin (hCG) and the early detection of intrauterine pregnancy by high resolution transvaginal ultrasound (TVS) have helped to enable the timely diagnosis of an extra-uterine pregnancy (1 -6 ).

Prior studies have shown the probability that creatine phosphokinase (CPK), an intracellular enzyme that catalyzes the formation of adenosine triphosphate (ATP) from creatine phosphate and adenosine diphosphate (ADP) might be practical in detecting an EP (1 -6 ). However, other investigations have shown that CPK is not useful in detecting EP (7 -16 ). Only two studies have evaluated the diagnostic value of CPK isoenzymes, a study by Kurzel et al. estimated CPK-MM levels and found a poor difference (14 ), Katsikis et al. (6 ) demonstrated that decreased CPK-MB has a relative ratio with the diagnosis of EP.

This research was planned to assess the predictive capability of CPK and its isoenzyme (CPKMB) in diagnosing an EP.

## Materials and Methods

In this prospective study, pregnant patients between 8-10 weeks of gestation, who presented to the emergency departments of Al-Zahra and Shahid Beheshti hospitals in Isfahan with abdominal pain, vaginal bleeding, or both were included in this study. The duration of the study was from February 2007 to September 2008. Exclusion criteria were: history of injury, muscle diseases, chronic renal failure, surgery, intramuscular injections, heart problems, hypothyroidism, respiratory distress, malignancies, and consumption of drugs (such as codeine, carbenoxolone, dexamethasone). General information was collected from the patients, and their blood was drawn and sent to the laboratory, immediately following venipuncture. Informed consent was signed by all patients. Patients were followed to determine their diagnoses and then were placed into either the EP or non-EP group. The diagnosis was made by either TVS (the standard test for EP), β-hCG concentration, or both. Patients were approached until there were 53 patients in the EP group and 53 in the non-EP group. The estimation of sample size was based on a study by Katsikis et al. (6 ). that reported a sensitivity of 82.5%, specificity of 95%, and PLR of 8.2. SPSS software version 10 was used for data analysis and determination of sensitivity, specificity, negative predictive value (NPV), positive predictive value (PPV), negative likelihood ratio (NLR) and positive likelihood ratio (PLR) for both CPK and CPK-MB serum levels. Relative operating characteristic (ROC) curves were plotted for both CPK and CPK-MB serum levels. The areas under the ROC curves (AUC) were calculated and compared with the AUC (0.5) of the non-diagnostic test.

## Results

The mean maternal age was 28.1 ± 4.76 years; for the EP group it was 28.62 ± 4.97 years; for the non-EP group, it was 27.58 ± 4.53 years. Based on the t test, the mean maternal age in these two groups have no positive significant diferences (p=0.263). According to the ROC curve, the cutoff point for CPK serum concentration for the diagnosis of EP was 61 IU/L, and for CPK-MB it was 15.6 IU/L (Table 1). On the basis of a chisquare test, there was a significant relationship between CPK level and the type of pregnancy (p=0.0001), as well as between the CPK-MB level and pregnancy type (Table 2, p=0.003).

**Table 1:** CPK serum levels


	CPK <61 IU/L	CPK >61 IU/L	Total

**EP Group (n) within group%**	16	37	53
	30.2	69.8	100
**Non-EP (n) within group %**	34	19	53
	64.2	35.8	100
**Total (n) within group %**	50	56	106
	47.2	52.8	100


**Table 2:** CPK-MB serum levels


	CPK <15.6 IU/L	CPK >15.6 IU/L	Total

**EP Group (n) within group%**	15	38	53
	28.3	71.7	100
**Non-EP (n) within group%**	30	23	53
	56.6	43.4	100
**Total (n) within group %**	45	61	106
	42.5	57.5	100


In this study, the CPK concentration of greater than 61 IU/L as the cutoff point for the diagnosis of EP had the following results: sensitivity (69.81%), specificity (64.15%), PPV (66.07%), NPV (68%), PLR (1.95), and NLR (0.49). The results for CPK-MB at a concentration greater than 15.6 IU/L as a cutoff point for the diagnosis of EP were: sensitivity (71.7%), specificity (56.6%), PPV (62.29%), NPV (66.7%), PLR (1.65), and NLR (0.5).

The ROC curve plotted for CPK showed that the AUC was 0.692, whereas for CPK-MB the AUC was 0.647. Based on the Chi-square test there was no difference between these two biochemical markers for the diagnosis of EP (p=0.517).

## Discussion

Blastocyst implantation and invasion in the fallopian tube, where there is no submucosal layer, results in damage to the muscular layer of the tube and thus an increase of CPK in the maternal serum. This mechanism was first thought to be helpful as an indicator for the early diagnosis of EP by Lavie et al. (1 ). This conclusion was further reiterated by other reports (2 -6 ). However, in contrast to these findings, Vandermolen and Borzelleca have proven that serum creatine kinase and its level in maternal serum cannot significantly predict EP (7 ), which have also been accepted by other researchers (8 -11 ). Katsikis et al. (6 ) measured the level of this enzyme and its isoenzyme and suggested that CPK-MB could be considered an early indicator of EP.

These controversies emerged us for this recent trial. TVS and serial β-hCG determination can be suggestive of EP, particularly when the gestational sac is not visualized inside the uterus. However, these tools are not the standard for diagnosing EP because of their PPV and NPV. The current study has found a significant CPK concentration of more than 61 IU/L to have a sensitivity of 69.81% and a specificity of 64.15% (with a PPV of 66.07% and an NPV of 68%). We also found an primary increase at serum CPK-MB levels greater than 15.6 IU/L have a 71.7% sensitivity, 56.6% specificity, PPV of 62.29% and NPV of 66.72%. These data are similar to the results of the above mentioned articles (1 -6 ), however better diagnostic tools are necessary.

## Conclusion

Considering the sensitivity and positivity of CPK and its isoenzyme CPK-M, it appears that laparoscopy remains the gold standard diagnostic tool for recognition and confirmation of EP.
